# Reliability of short-term measurements of heart rate variability (HRV) in patients with non-permanent atrial fibrillation

**DOI:** 10.1038/s41598-026-64135-1

**Published:** 2026-07-29

**Authors:** Erlend Flo Lenz, Andreas Berg Sellevold, Jon Magne Letnes, Bjarne Martens Nes, Jan Pål Loennechen

**Affiliations:** 1 Department of Circulation and Medical Imaging, Norwegian University of Science and Technology, Trondheim, Norway; 2 Department of Cardiology and Cardiothoracic Surgery, St. Olavs University Hospital, Trondheim, Norway

**Keywords:** Heart rate variability, HRV, Reliability, Short-term recordings, Atrial fibrillation, Cardiology, Diseases, Medical research

## Abstract

Short-term measurements of heart rate variability (HRV) are practical, noninvasive markers of autonomic activity, and reduced HRV has been associated with onset and recurrence of non-permanent atrial fibrillation. However, clinical use is limited by strict standardization requirements and uncertain reliability in clinical populations. We evaluated the reliability of short-term HRV derived from 48-h Holter recordings during sinus rhythm in patients with non-permanent AF. Fifty participants underwent two standardized outpatient recordings at Holter attachment and removal, and additional, manually selected, out-of-hospital daytime and night-time presumed-sleep segments, with low editing burden, were extracted. HRV was assessed using SDNN and RMSSD. Reliability was evaluated using Bland-Altman limits of agreement (LoA) and intraclass correlation coefficients (ICC). For standardized outpatient recordings, LoA ranged from 43 to 242% for SDNN and 38% to 280% for RMSSD, with ICC values of 0.67 and 0.43, respectively. Consecutive night-time presumed-sleep segments showed higher relative reliability, with ICC values of 0.79 for SDNN and 0.89 for RMSSD, but absolute agreement remained limited (LoA 53%-173% and 59%-170%). These findings indicate poor absolute reliability in this workflow, limiting individual-level clinical interpretation, and likely reflecting a combination of measurement-process variability, inherent to a clinically pragmatic workflow and biological within-person variability in HRV.

**Supplementary Information:**

The online version contains supplementary material available at https://doi.org/10.1038/s41598-026-64135-1.

## Introduction

Heart rate variability (HRV) is the measured variability of the RR-interval length, only using cardiac beats originating from the sinoatrial node, defined as NN-intervals^1^. It is a noninvasive, indirect measurement, which is used for assessing the activity of the autonomic nervous system^2,3^.

Decreased HRV is commonly interpreted as a sign of autonomic imbalance, indicating a weakened capacity for adaptation and a reduced ability in physiological systems to maintain homeostasis^4,5^. Several studies demonstrate an association between reduced HRV and an unfavorable prognosis in patients with heart diseases^6–8^.

The autonomic nervous system is important in the development, sustenance and recurrence of atrial fibrillation (AF)^9,10^. Recurrence after treatments aimed at restoring sinus rhythm, such as cardioversion and ablation, remains a major challenge^11^. Reduced HRV has been shown to be a predictor for both onset and post-treatment recurrence in patients with non-permanent AF^12–14^.

Despite its clinical potential, HRV is still mostly used in research settings^15^. Short-term (ST) time-domain measurements^16^, such as the standard deviation of normal-to-normal intervals (SDNN) and the root mean square of successive differences (RMSSD), offer practical benefits and are widely used in research. Although practical due to feasibility, time-efficient measurements and easier standardization, its use as a reliable marker has been questioned^15,17,18^.

An important limitation of ST-HRV is its dependence on standardized recording and processing conditions. Both patient-related factors, such as posture, time of day, recent activity, respiration, caffeine or nicotine intake, and medication timing, and signal- or processing-related factors, such as artifact handling, segment selection, and HRV calculation, may influence measured HRV^19–27^. This makes extensive standardization and processing difficult to achieve in routine clinical practice. Long-term ECG recordings, which are already widely used in cardiology, may provide a clinically pragmatic opportunity to extract short sinus-rhythm segments for HRV analysis without requiring a separate standardized HRV examination. However, the reproducibility of both standardized ST-HRV measurements and Holter-derived ST-HRV segments remains insufficiently established in clinical populations^17,18^.

The aim of this study was to assess the reliability of a clinically pragmatic workflow for short-term HRV measurements derived from long-term ECG recordings during sinus rhythm in patients with non-permanent AF. The primary objective was to evaluate whether a standardized outpatient ST-HRV measurement was reproducible after 48 hours. Secondary objectives were to assess whether out-of-hospital daytime and night-time presumed-sleep ST-HRV segments were comparable to standardized outpatient measurements, and to evaluate the reproducibility of two consecutive night-time presumed-sleep segments.

## Material and methods

### Study design, NEXAF Trial and authorization

This observational reliability study was conducted as a substudy of the Norwegian Exercise in Atrial Fibrillation Trial (NEXAF Trial)^28^, a multicenter randomized controlled trial investigating the effects of one year of systematic exercise compared with usual care in patients with non-permanent AF. The NEXAF Trial is registered at ClinicalTrials.gov (NCT05164718). The present substudy was approved by the NEXAF steering group and was conducted within the ethical approval granted for the NEXAF Trial by the Regional Committee for Medical and Health Research Ethics in Central Norway (REK 213,848). All participants received written and oral information about the HRV substudy before Holter attachment and provided written informed consent for participation and additional analyses related to this substudy. The signed consent forms are archived at the study site.

### Sample size rationale

The target sample size was defined a priori. Because the primary objective was to assess agreement and reliability rather than a between-group difference, the sample size rationale was based on Bland-Altman agreement analysis. Power calculations were performed using PASS 2020 for samples of 50 and 70 paired observations. Agreement was defined as the 95% confidence intervals around the Bland-Altman limits of agreement falling within a prespecified maximum allowable difference. We evaluated maximum allowable differences of ± 10 and ± 15 ms, anticipated mean differences of 1, 3, and 5 ms, and anticipated standard deviations of the paired differences of 3 and 5 ms. Under the most favorable prespecified scenario, with a maximum allowable difference of ± 10 ms, an anticipated mean difference of 1 ms, and an anticipated SD of 3 ms, 50 paired observations provided 98.7% power to demonstrate agreement. Under a less favorable but still acceptable scenario, with a maximum allowable difference of ± 15 ms and an anticipated SD of 5 ms, 50 paired observations provided 92.2% power. Increasing the sample size from 50 to 70 provided limited additional gain in scenarios with clearly good or clearly poor agreement. A sample of 50 participants was therefore considered sufficient for the primary reliability objective, while acknowledging that this sample size limits the precision of the estimated limits of agreement.

### Study population

Participants in the present study were recruited consecutively from the Trondheim site of the NEXAF Trial after completion of the one-year intervention or control period. Key inclusion criteria for the NEXAF Trial were a diagnosis of non-permanent AF in hospital registries, limited vigorous or moderate intensity exercise for the last three months, and age between 18 and 79 years. AF-related exclusion criteria included one continuous AF-episode of ≥ 3 months during the last year, recent or planned ablation, or AF as a complication to surgery, infection or acute coronary syndromes. Patients with unstable coronary heart disease, de-compensated heart failure, left ventricular ejection fraction < 40%, significant valvular pathology, moderate to severe chronic obstructive pulmonary disease, ongoing severe cancer or active cancer treatment, psychiatric/cognitive issues that hinder compliance or contraindications to exercise were excluded.

For the present substudy, exclusion criteria otherwise aligned with those of the NEXAF Trial. The study-specific requirement was sinus rhythm during the HRV recordings included in the analyses. No additional exclusion criteria were applied based on variables known to influence HRV, such as medication use, comorbidity, sex, age, or physical capacity, because the study was designed to compare repeated HRV measurements within the same individuals.

### Data collection and ECG recordings

Data were collected using 48-h long-term ECG recordings with a Philips Zymed Digitrak XT Holter Recorder, with a sampling frequency of 175 Hz. Recordings were performed between March 15, 2023, and April 17, 2024. Electrode placement, recording initiation, device removal, and upload to Holter 2010 Plus software were performed by the same cardiology research nurse trained in long-term ECG monitoring.

Holter attachment took place at the outpatient clinic of the Department of Cardiology, St. Olav’s Hospital, Trondheim, Norway, between 08:00 and 14:30. Usual medications were continued, and participants were instructed to abstain from caffeine and nicotine for at least one hour before each standardized recording. After 15-30 min of seated rest, electrodes were applied. Participants then remained seated in a standardized position with legs at a 90-degree angle, hands resting on thighs, and spontaneous breathing. The first standardized 5-min ECG segment was recorded at Holter attachment. Participants were subsequently instructed to live as normally as possible during the 48-h recording period, without specific activity or dietary restrictions apart from the instructions given before standardized recordings. At device removal, a second standardized 5-min recording was performed using the same procedure before terminating the recording.

### ECG segment selection and quality control

For each participant, five 5-min ECG segments were selected from the 48-h Holter recording: two standardized outpatient segments, one out-of-hospital daytime segment, and two consecutive night-time presumed-sleep segments.

The standardized outpatient segments were defined by the recorded start times at Holter attachment and removal. These represented fixed clinical measurements and were not replaced by alternative windows if signal quality was suboptimal or ectopic activity was present. If a standardized segment was invalid because of AF or insufficient signal quality, it was treated as missing for analyses requiring that segment.

The out-of-hospital daytime segment was selected from the recording period before 20:00 on the day of Holter attachment or the following day. This segment was not controlled for posture, physical activity, food intake, or other behavioral factors. Candidate windows were identified during manual review by scrolling through the Holter recording within the predefined time interval. A segment was selected if it showed sinus rhythm, visually acceptable signal quality, and low editing burden. No formal algorithmic selection, activity log, or prespecified preference for exact clock time within the interval was used.

Night-time presumed-sleep segments were selected between 00:00 and 06:00 on the night after Holter attachment or the following night. Presumed sleep was defined by clock time only; sleep was not verified by sleep diary, activity log, actigraphy, or polysomnography. During manual review, a 10-min period with sinus rhythm and visually acceptable signal quality was identified and divided into two consecutive 5-min segments. If either 5-min segment failed to meet the eligibility criteria, the entire 10-min period was rejected and another eligible period was sought within the predefined time interval.

All selected segments were required to contain sinus rhythm and no AF. Signal quality was assessed visually in Holter 2010 Plus. For non-standardized out-of-hospital daytime and night-time presumed-sleep segments, candidate windows were selected to keep the proportion of beats requiring manual invalidation during NN editing at or below 2% where possible. This threshold was not applied to the standardized outpatient segments, because these represented fixed clinical measurements.

### NN editing and HRV computation

All 5-min ECG segments were reviewed and edited in Holter 2010 Plus by the same author. Beats automatically classified by the software as abnormal were visually reviewed. Beats considered to be sinus beats despite automatic abnormal classification were corrected, whereas non-sinus beats, ectopic beats, and artifacts were manually marked as invalid. When a beat was marked as invalid, the RR interval before and after that beat was excluded from the HRV calculation by the Holter software.

The shortest and longest RR intervals in each 5-min segment were also inspected using the RR-interval plot to identify possible undetected non-sinus beats or artifacts. No manual interpolation was performed by the investigators. After editing, SDNN and RMSSD values were obtained directly from the Holter 2010 Plus report and entered into the study dataset.

### Missing data

Missing HRV data occurred when ECG recordings or specific segments were unavailable or invalid because of AF, insufficient signal quality, or incomplete recording. No imputation was performed. Reliability analyses were conducted as available-case analyses for each comparison, meaning that a participant was included in a given analysis only if both measurements required for that comparison were available and valid. The number of participants included is reported for each analysis.

### Statistical analysis

All statistical analyses were performed using Python version 3.12. Data handling and descriptive analyses were performed using pandas and NumPy, Bland-Altman analyses were implemented using custom Python code with SciPy, intraclass correlation coefficients were calculated using pingouin, and figures were generated using matplotlib.

Absolute reliability was assessed using Bland-Altman plots and 95% limits of agreement (LoA). Bias was calculated as the mean difference between paired measurements on the original millisecond scale. Because Bland-Altman plots indicated heteroscedasticity, LoA were calculated after ln-transformation and then back-transformed to percentages. Relative reliability was assessed using intraclass correlation coefficients (ICC) from a two-way mixed-effects consistency model.

Because night-time presumed-sleep segments were defined by clock time and not by objective sleep assessment, post hoc sensitivity analyses were performed using heart rate as a proxy for a lower-activity or sleep-like state (Supplementary Table S1). For these analyses, the mean heart rate of the two night-time presumed-sleep segments was compared with the heart rate of the out-of-hospital daytime segment. Sensitivity analysis A included only participants whose mean night-time presumed-sleep heart rate was lower than their daytime heart rate. Sensitivity analysis B5 included only participants with a daytime-to-night-time heart rate reduction of at least 5 beats per minute, and sensitivity analysis B10 included only participants with a reduction of at least 10 beats per minute.

## Results

### Descriptive statistics

Fifty participants were included in the study. General characteristics of the population are described in Table 1. Mean age was 66.6 (range 47-79) years, 38% were women and 22% were obese (BMI > 30 kg/m^2^). Mean VO_2max_ was 32.7 mL/kg/min for men and 26.6 mL/kg/min for women. Ten participants had other heart disease or diabetes and 68% of the participants used either beta blockers or other antiarrhythmics/antihypertensives.

**Table 1:** General characteristics of the study population.

Characteristic	
Age (years)	66.6 (9)
Women	19 (38%)
BMI (kg/m^2^)	28.3 (5.6)
VO_2 max_ (mL/kg/min)	30.3 (7.2)
Persistent AF	15 (30%)
Other heart disease	10 (20%)
Earlier myocardial infarction	10 (20%)
Compensated heart failure, EF > 40%	5 (10%)
Diabetes mellitus (n) (Type 1/Type 2)	1/0
Beta blockers	19 (38%)
Flecainide	5 (10%)
Antihypertensive medication*	26 (52%)
ACE inhibitor/ARBs	21 (42%)
Calcium channel blockers	9 (18%)
Thiazide diuretics	7 (14%)

Values are described as mean value (± SD) or n (%), unless otherwise specified. Abbreviations: SD: Standard deviation, BMI: Body Mass Index, AF: Atrial Fibrillation, EF: ejection fraction, ACE: Angiotensin-converting enzyme, ARBs: Angiotensin receptor blockers, *: not included beta blockers.

**Fig. 1:**
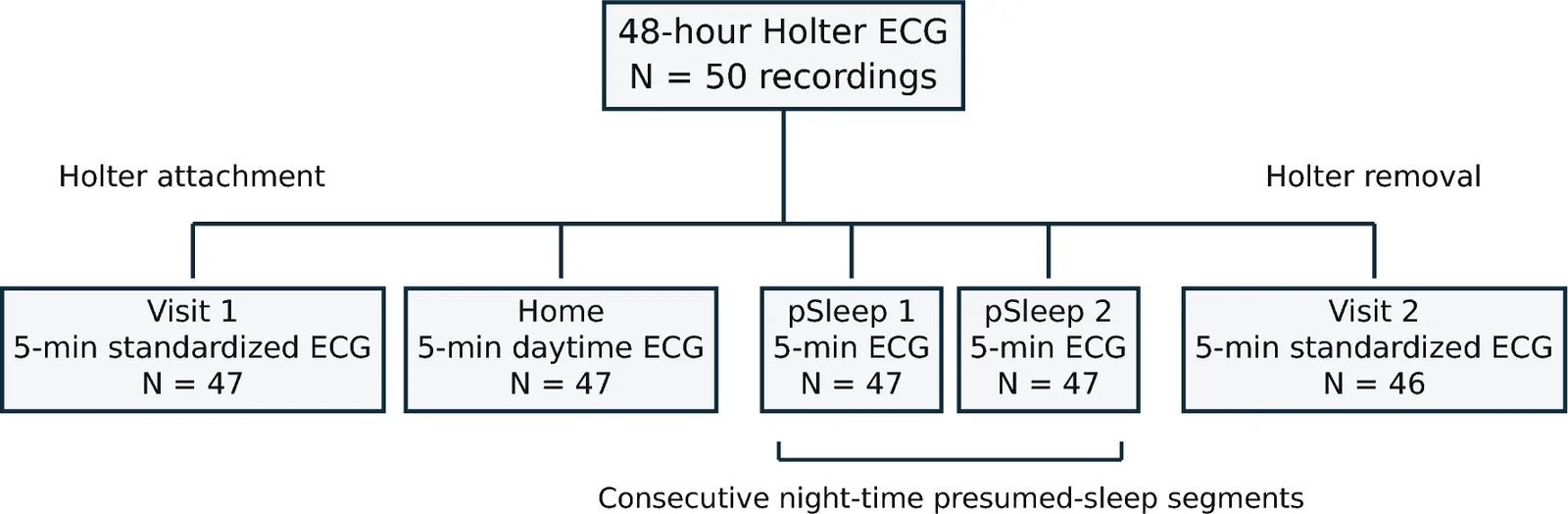
Overview of 5-min ECG segments extracted from the 48-h Holter ECG recording. Five 5-min ECG segments were extracted from each participant’s 48-h Holter ECG recording. Visit 1 and Visit 2 were standardized outpatient recordings performed at Holter attachment and removal. Home was an out-of-hospital daytime segment. pSleep denotes night-time presumed sleep; pSleep 1 and pSleep 2 were consecutive 5-min segments selected from a predefined nocturnal time window without objective sleep verification. N indicates the number of included segments available for each measurement.

A total of fifty 48-h ECG-recordings were collected. Seventeen recordings had at least one episode of AF, with eleven lasting less than five minutes of the total 48-h. Three recordings were deemed invalid due to AF during most of the recording. Additionally, one of the recordings was discontinued after 36 h, and no controlled outpatient recording was conducted at disconnection. Editing burden was low overall, with a higher burden in three participants, and is reported in Supplementary Table S2. The number of included recordings is detailed in Fig. 1, while descriptive results for the various HRV-parameters of the included recordings are provided in Table 2.

**Table 2:** Descriptive results with mean value for heart rate, SDNN and RMSSD for all recordings.

Measurement	Heart rate (SD) (Min, Max)	SDNN (SD) (Min, Max)	RMSSD (SD) (Min, Max)
Visit 1	65.4 bpm (15 bpm) (39 bpm, 135 bpm)	39.2 ms (21.4 ms) (8.6 ms, 103.1 ms)	32.0 ms (17.5 ms) (9.0 ms, 92.5 ms)
Visit 2	65.8 bpm (9.7 bpm) (51 bpm, 89 bpm)	41.2 ms (28.7 ms) (12.1 ms, 129.2 ms)	38.3 ms (42.4 ms) (7.8 ms, 225.2 ms)
pSleep 1	55.1 bpm (6.7 bpm) (42 bpm, 75 bpm)	51.2 ms (26.1 ms) (17.2 ms, 111.9 ms)	41.9 ms (25.6 ms) (9.7 ms, 117.4 ms)
pSleep 2	55.2 bpm (6.8 bpm) (43 bpm, 75 bpm)	54.6 ms (32.3 ms) (15.2 ms, 154.3 ms)	41.6 ms (25.5 ms) (12.1 ms, 110.9 ms)
Home	63.1 bpm (6.8 bpm) (50 bpm, 77 bpm)	41.1 ms (18.5 ms) (17.2 ms, 101.2 ms)	34.3 ms (17.9 ms) (7.8 ms, 97.8 ms)

Values are presented as mean (SD) (min, max). SD, standard deviation; SDNN, standard deviation of normal-to-normal intervals; RMSSD, root mean square of successive normal-to-normal interval differences; bpm, beats per minute; pSleep, night-time presumed sleep.

### Absolute reliability

In the comparison between the standardized outpatient recordings (Visit 1 vs Visit 2) for RMSSD, a bias of—6.0 ms (95% CI,—16.3 ms to 4.3 ms) was observed, with LoA spanning from 38 to 280% (Table 3). For the two successive recordings during night-time presumed-sleep (pSleep 1 and 2), RMSSD displayed a bias of 0.3 ms (95% CI,—3.1 ms to 3.7 ms), with LoA ranging from 59 to 170% (Table 3).

**Table 3:** Reliability analysis for 5-min HRV-measurements with calculated biases, limits of agreement (LoA) and intraclass correlation coefficient (ICC) values between measurements.

Measurements compared		Parameter	Range	Bias (95% CI)	Lower LoA (95% CI)	Upper LoA (95% CI)	ICC (95% CI)
Visit 1	Visit 2	SDNN	- 72.2 ms, 42.9 ms	**- 1.7 ms** (- 7.8 ms, 4.4 ms)	**43%** (34%, 54%)	**242%** (193%, 303%)	**0.67** (0.47, 0.80)
		RMSSD	- 184.7 ms, 26.9 ms	**- 6.0 ms** (- 16.3 ms, 4.3 ms)	**38%** (29%, 49%)	**280%** (215%, 364%)	**0.43** (0.16, 0.64)
pSleep 1	pSleep 2	SDNN	- 61.5 ms, 48.2 ms	**- 3.4 ms** (- 9.0 ms, 2.2 ms)	**53%** (46%, 62%)	**173%** (149%, 201%)	**0.79** (0.65, 0.88)
		RMSSD	- 31.3 ms, 41.7 ms	**0.3 ms** (- 3.1 ms, 3.7 ms)	**59%** (51%, 68%)	**170%** (148%, 195%)	**0.89** (0.82, 0.94)
Visit 1	Home	SDNN	- 60.6 ms, 45.6 ms	**- 1.9 ms** (- 7.4 ms, 3.6 ms)	**47%** (38%, 59%)	**254%** (204%, 316%)	**0.56** (0.33, 0.73)
		RMSSD	- 65.3 ms, 37.5 ms	**- 2.3 ms** (- 6.4 ms, 1.9 ms)	**46%** (39%, 55%)	**181%** (152%, 217%)	**0.68** (0.49, 0.81)
Visit 1	pSleep 1	SDNN	- 74.8 ms, 43.2 ms	**- 12.0 ms** (- 19.8 ms,- 4.2 ms)	**26%** (20%, 34%)	**224%** (170%, 297%)	**0.39** (0.12, 0.61)
		RMSSD	- 59.0 ms, 21.2 ms	**- 9.9 ms** (- 15.6 ms,- 4.1 ms)	**29%** (23%, 38%)	**216%** (167%, 280%)	**0.60** (0.38, 0.76)

CI, confidence interval; LoA, limits of agreement; ICC, intraclass correlation coefficient; SDNN, standard deviation of normal-to-normal intervals; RMSSD, root mean square of successive normal-to-normal interval differences; pSleep, night-time presumed sleep.

When comparing the standardized outpatient recording (Visit 1) with the home recording (Home), the RMSSD bias was – 2.3 ms (95% CI,—6.4 ms to 1.9 ms), with lower and upper LoA 46% and 181% respectively. The recording Visit 1 against pSleep 1 showed a bias of—9.9 ms (95% CI,—15.6 ms to—4.1 ms) lower LoA 29% and upper LoA 216% for RMSSD (Table 3). SDNN values showed similar magnitudes of bias and LoA as those measured for RMSSD, for all the comparisons (Table 3).

**Fig. 2:**
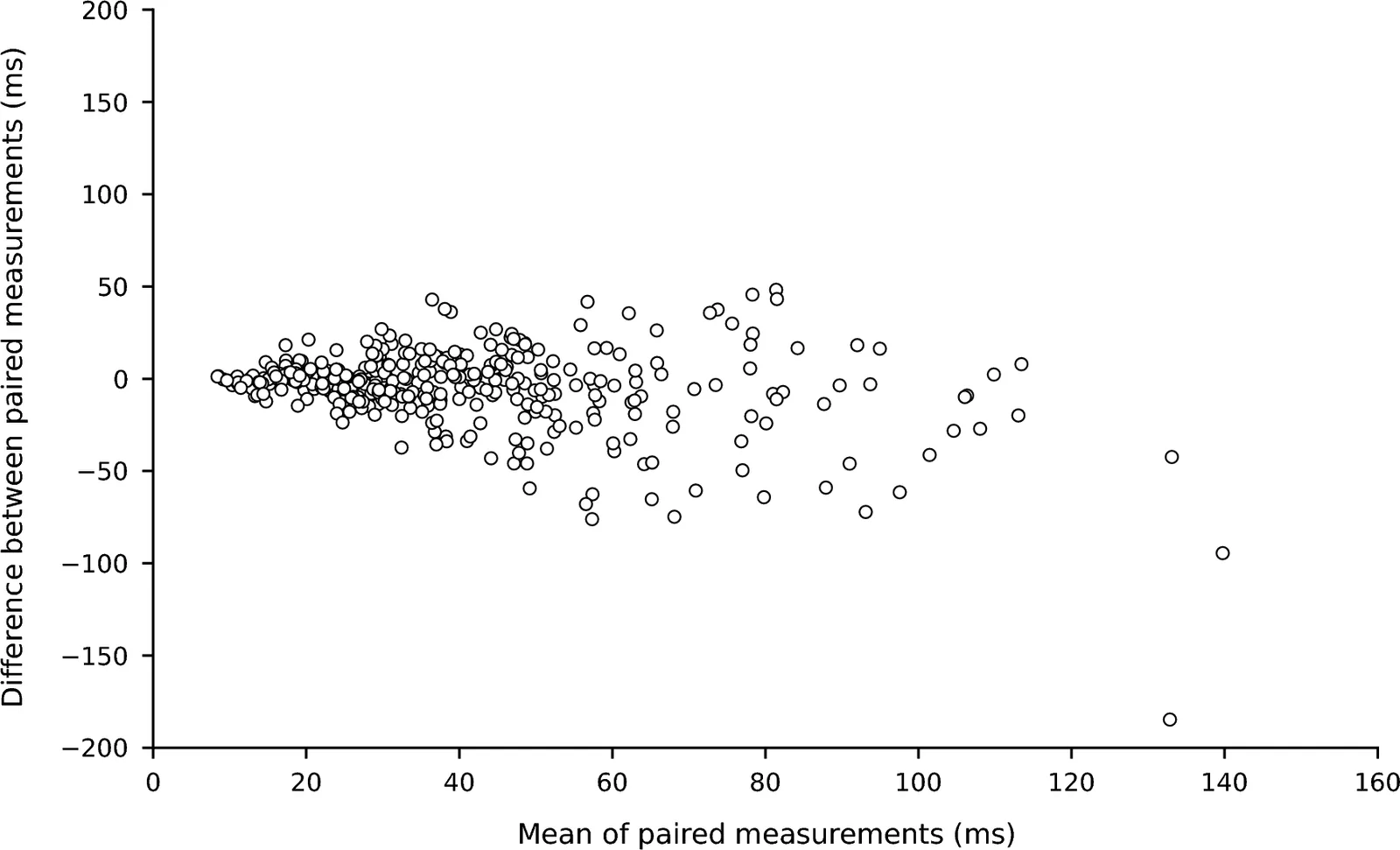
Bland–Altman plot of all short-term HRV measurements across the included comparisons to visualize heteroscedasticity. The plot includes paired SDNN and RMSSD measurements from Visit 1 vs Visit 2, Visit 1 vs Home, Visit 1 vs pSleep 1, and pSleep 1 vs pSleep 2. The mean of the paired measurements is plotted on the x-axis and the difference between the paired measurements on the y-axis. pSleep, night-time presumed sleep.

All the measurements showed signs of heteroscedasticity by visual inspection of the Bland-Altman plots, both for SDNN and RMSSD. In other words, speaking to the intrinsic property of the HRV measure, the higher the variability measured, the larger the difference between measurement 1 and 2 (Fig. 2). Some outliers in either measurement 1 or 2 (Fig. 2) impacted reproducibility between measurement 1 and 2 across all measurements (Table 3).

### Relative reliability

ICC values ranged from 0.39 to 0.89 (Table 3). For the comparison between the standardized outpatient recordings (Visit 1 vs Visit 2), ICC values of 0.67 for SDNN and 0.43 for RMSSD were observed (Table 3). The two successive night-time presumed-sleep-recordings displayed the highest ICC values for both SDNN, ICC 0.79, and RMSSD, ICC 0.89.

When comparing the standardized outpatient recording (Visit 1) vs. the home recording, SDNN showed an ICC value of 0.56, while RMSSD showed an ICC value of 0.68 (Table 3). Visit 1 vs pSleep 1 displayed an ICC value of 0.39 for SDNN and a value of 0.60 for RMSSD.

### Post hocsensitivity analysis

Post hoc HR-based sensitivity analyses of the consecutive night-time presumed-sleep segments did not materially change the interpretation of the primary analysis. Restricting the analyses to participants with lower night-time than daytime heart rate, or to those with ≥ 5 bpm or ≥ 10 bpm daytime-to-night-time heart rate reduction, showed the same overall pattern, with relatively high ICC values but persistently wide limits of agreement (Supplementary Table S1).

## Discussion

We found wide LoA for all the recordings, small and negligible biases for most recordings and ICC values ranging from 0.39 to 0.89, indicating poor absolute reliability across all measurements and varying relative reliability. The two standardized outpatient recordings showed the widest LoA and the worst relative reliability, while the two successive night-time presumed-sleep recordings demonstrated the narrowest LoA and the best relative reliability. The poor absolute reliability observed in this workflow likely reflects a combination of measurement-process variability and biological intraindividual variability in HRV.

The present study was designed to evaluate a clinically pragmatic workflow for extracting short-term HRV from long-term ECG recordings during sinus rhythm. Consequently, the observed variability should not be interpreted as reflecting physiological HRV variability alone. Rather, the reliability estimates represent the combined influence of biological within-person variability and measurement-process variability related to clinical standardization, segment selection, signal quality, rhythm classification, sleep-state classification, and manual NN editing. This distinction is important when considering whether Holter-derived ST-HRV can be used as an individual clinical marker^20^.

One would expect factors influencing HRV, including activity, posture, and other autonomic stimuli, to be more stable during two consecutive night-time presumed-sleep segments than between recordings separated by hours or days. The improved relative reliability and narrower limits of agreement for the consecutive night-time presumed-sleep segments is consistent with this. However, the absolute reliability remained poor even for these consecutive segments, indicating that the variability was still substantial. Because the night-time presumed-sleep segments were manually selected for visually acceptable signal quality and low editing burden, the improved reliability may, in part, reflect a selection bias arising from this non-random selection. Notably, the editing burden of these segments was low and comparable to that of the standardized outpatient segments, which were not selected on signal quality, suggesting that selection for low editing burden is unlikely to have contributed to the improved reliability (Supplementary Table S2). Nevertheless, selection bias related to other, unmeasured characteristics of the manually chosen windows cannot be fully excluded.

The clinical interpretation of HRV change is challenging because no established minimal clinically important difference exists for short-term SDNN or RMSSD in patients with non-permanent AF. However, if ST-HRV were to be used for individual clinical follow-up, the expected measurement variation would need to be smaller than the change considered clinically meaningful. Using a typical 5-min value of approximately 50 ms for SDNN reported in healthy populations^29^, even a relatively large hypothetical change of 25% would correspond to only 12.5 ms. Our findings indicate that, in 95% of cases, a repeated SDNN value could range from approximately 43% to 242% of the first value. For example, a typical SDNN value of 50 ms^29^ could be measured as approximately 22 ms or 121 ms two days later, without necessarily representing a true clinical change. This observed variation is substantially larger than the hypothetical 12.5 ms change used in this example.

These differences in measured HRV, despite standardized outpatient recording conditions, are likely to reflect both methodological variability inherent to a clinically feasible workflow and HRV’s sensitivity to intrinsic and extrinsic physiological influences. Within this population and workflow, this degree of variability limits the usefulness of ST-HRV for individual-level interpretation in routine clinical practice. The low-to-moderate ICC values for the repeated standardized outpatient measurements also question whether this workflow can reliably distinguish between individuals based on HRV-derived autonomic information.

Although several studies on the reliability of ST-HRV have been conducted over the past 20 years, the overall understanding remains largely unchanged, and can be summarized as follows: (i) HRV tends to be less reliable in clinical populations compared to healthy individuals^18^; (ii) the reliability for different clinical populations is poorly investigated^18^; (iii) multiple studies show poor absolute reliability despite excellent relative reliability^18,30–33^.

Few rigorous studies have examined the reliability of standardized ST-HRV in clinical populations; however, the findings of this study align well with similar research^31,34^, which also reported substantial day-to-day variability within individuals. Maestri et al. reported 95% LoA ranging from 0.59 to 1.61 for SDNN and from 0.47 to 2.14 for RMSSD, along with ICC values of 0.82 for SDNN and 0.76 for RMSSD^31^. Our study showed wider limits of agreement and lower ICC values for the standardized outpatient recordings. This difference may be explained by both biological and methodological factors, including differences in study population, degree of standardization, recording conditions, and handling of outliers.

We were cautious about excluding outliers, as extreme values may represent true measurements encountered in clinical practice. Excluding such observations could artificially improve reliability estimates and reduce the clinical relevance of a pragmatic reliability study. At the same time, outlying values may also reflect measurement-process variability^22,23^, including segment quality, ectopy, or manual editing. The decision not to exclude outliers therefore supports a pragmatic interpretation of the results^20^, but also reinforces that the observed variability reflects the full measurement workflow rather than biological HRV variability alone.

## Strengths and limitations

A major strength of this study is that it evaluates a clinically pragmatic workflow for extracting short-term HRV from long-term ECG recordings in a relevant clinical population with non-permanent AF. This reflects a potential clinical implementation context, where HRV would be derived from Holter recordings already performed in routine cardiology rather than from idealized laboratory recordings. The study also assessed both absolute and relative reliability, included repeated standardized outpatient recordings, and evaluated out-of-hospital daytime and night-time presumed-sleep segments. In addition, post hoc sensitivity analyses were performed to assess the robustness of the night-time presumed-sleep findings using heart rate as a proxy for a lower-activity or sleep-like state.

This pragmatic design introduces important limitations. Participant behavior was not strictly controlled before or during the 48-h recordings. Medication timing, recent activity, meals, respiration, sleep, and posture outside the standardized outpatient recordings may have influenced HRV^20,22^. Although this reflects routine clinical conditions, it also means that the observed variability may partly reflect limited standardization. The findings are also limited to patients with non-permanent AF in sinus rhythm and may not be generalizable to healthy individuals, other cardiac populations, patients with permanent AF, or other ECG systems and HRV workflows.

Segment selection represents a major limitation^26,27^. Out-of-hospital daytime and night-time presumed-sleep segments were selected manually and non-randomly during visual review rather than by a fully algorithmic procedure. Although predefined time windows and eligibility criteria were used, the number of screened candidate windows and the number of rejected windows were not prospectively stored. Selection was therefore quality- and low-editing-driven, and selection or curation bias cannot be quantified or excluded. This may have biased reliability estimates toward better agreement, independent of editing burden. Consequently, the improved reliability observed for night-time presumed-sleep segments cannot be attributed to physiological stability alone, and reliability under less curated ambulatory conditions may be no better.

Night-time presumed-sleep segments were defined by clock time only. Sleep was not confirmed by sleep diary, activity log, actigraphy, or polysomnography. Consequently, these segments may include quiet wakefulness, nocturnal awakenings, insomnia, or other night-time conditions. The HR-based sensitivity analyses partly address this limitation by evaluating whether results were robust among participants with lower night-time heart rate, but heart rate is only an indirect proxy and cannot verify sleep state.

Manual NN editing is another major limitation and potential source of measurement-process variability^21^. All 5-min segments were reviewed and edited by one author, no manual interpolation was performed and inter- or intra-rater reliability for editing were not quantified. Thus, we cannot exclude the relative contribution of editing, ectopy/noise, and biological variability. Furthermore, the Holter sampling frequency of 175 Hz may influence short-term HRV accuracy, particularly for beat-to-beat measures such as RMSSD, and HRV values were obtained from Holter 2010 Plus software, which may limit reproducibility across other systems.

Finally, although the sample size of 50 participants was justified a priori for the primary reliability objective, it limits the precision of the estimated limits of agreement and their confidence intervals. The Bland-Altman plots indicated heteroscedasticity, with larger differences at higher HRV values. Although log-transformed limits of agreement were used to address proportional error, this variability complicates individual clinical interpretation. Furthermore, because the standardized outpatient recordings themselves showed poor absolute reliability, comparisons with out-of-hospital daytime or night-time presumed-sleep segments cannot establish interchangeability and should be interpreted cautiously^35^. ICC values should also be interpreted cautiously, as they depend on total population variation and may be elevated in HRV studies because of the large natural between-person variation in HRV values^29,36^.

## Conclusion

Short-term HRV measurements from predefined standardized outpatient segments and manually non-randomly selected out-of-hospital sinus-rhythm segments of 48-h Holter recordings showed poor absolute reliability and inconsistent relative reliability in this workflow and population with non-permanent atrial fibrillation. Consecutive night-time presumed-sleep segments showed better relative reliability than standardized outpatient recordings, but absolute agreement remained insufficient for confident individual-level interpretation. The observed variability likely reflects the combined influence of measurement-process variability inherent to a clinically pragmatic Holter-derived ST-HRV workflow and biological within-person variability. Because the standardized outpatient recordings themselves showed poor absolute reliability, comparisons with out-of-hospital daytime and night-time presumed-sleep segments cannot establish interchangeability and should be interpreted cautiously.

## Supplementary Information

Supplementary Information.

## Data Availability

The datasets generated and/or analyzed during the current study are not publicly available due to legal and ethical restrictions on sharing sensitive personal data. De-identified data may be available from the corresponding author on reasonable request, subject to institutional approvals and a data access agreement.

## Abbreviations

HRV: Heart rate variability
AF: Atrial fibrillation
SDNN: Standard deviation of normal-to-normal intervals
RMSSD: Root mean square of successive differences
ST: Short term
NEXAF: Norwegian exercise in atrial fibrillation trial
LoA: Limits of agreement
ICC: Intraclass correlation coefficient
CI: Confidence interval

## Code availability

The Python code used for the statistical analyses is available from the corresponding author upon reasonable request. The code does not include patient-level data.
