# Supplementary material for: Reliability of short-term measurements of heart rate variability (HRV) in patients with non-permanent atrial fibrillation
Source: Sci Rep. 2026 Jul 29;16:27938. doi: 10.1038/s41598-026-64135-1 (PMC13550427; doi:10.1038/s41598-026-64135-1)
Supplement: Supplementary file 1 [file 41598_2026_64135_MOESM1_ESM.pdf]

Supplementary Information

Reliability of short-term measurements of heart rate variability (HRV) in patients with non-permanent atrial fibrillation

Erlend Flo Lenz, Andreas Berg Sellevold, Jon Magne Letnes, Bjarne Martens Nes, Jan Pål Loennechen

Supplementary Table S1. Sensitivity analyses of absolute and relative reliability for consecutive night-time presumed-sleep HRV segments.

Analysis	Measure	N	Mean difference, ms (95% CI)	SD difference, ms	Lower LoA, % (95% CI)	Upper LoA, % (95% CI)	Single ICC (95% CI)	Average ICC (95% CI)
Original dataset	SDNN	47	-3.4 (-9.0–2.2)	19.1	53.0 (46.0–62.0)	174.0 (150.0–203.0)	0.79 (0.65–0.88)	0.88 (0.79–0.93)
Original dataset	RMSSD	47	0.3 (-3.1–3.8)	11.7	59.0 (51.0–68.0)	171.0 (149.0–196.0)	0.89 (0.82–0.94)	0.94 (0.90–0.97)
Sensitivity analysis A	SDNN	44	-3.4 (-9.4–2.5)	19.6	54.0 (46.0–63.0)	176.0 (150.0–206.0)	0.78 (0.63–0.87)	0.88 (0.77–0.93)
Sensitivity analysis A	RMSSD	44	0.5 (-3.2–4.2)	12.1	59.0 (51.0–68.0)	174.0 (150.0–201.0)	0.89 (0.80–0.94)	0.94 (0.89–0.97)
Sensitivity analysis B5	SDNN	35	-4.6 (-10.7–1.5)	17.8	58.0 (49.0–68.0)	164.0 (140.0–193.0)	0.84 (0.70–0.91)	0.91 (0.82–0.95)
Sensitivity analysis B5	RMSSD	35	0.5 (-3.2–4.3)	10.9	66.0 (58.0–75.0)	160.0 (140.0–183.0)	0.92 (0.85–0.96)	0.96 (0.92–0.98)
Sensitivity analysis B10	SDNN	17	-5.9 (-14.6–2.8)	16.9	56.0 (44.0–71.0)	160.0 (126.0–204.0)	0.88 (0.71–0.96)	0.94 (0.83–0.98)
Sensitivity analysis B10	RMSSD	17	-1.6 (-6.4–3.2)	9.3	66.0 (55.0–79.0)	148.0 (123.0–177.0)	0.95 (0.88–0.98)	0.98 (0.93–0.99)

All analyses compare pSleep 1 with pSleep 2. Sensitivity analysis A included participants whose mean night-time presumed-sleep heart rate was lower than daytime heart rate. Sensitivity analyses B5 and B10 included participants with at least 5 and 10 bpm lower mean night-time presumed-sleep heart rate than daytime heart rate, respectively.

Difference was calculated as pSleep 1 minus pSleep 2. Percentage limits of agreement were calculated from log-transformed paired differences and back-transformed to percentages. ICC was calculated using a two-way mixed-effects consistency model.

LoA, limits of agreement; ICC, intraclass correlation coefficient; CI, confidence interval; SDNN, standard deviation of normal-to-normal intervals; RMSSD, root mean square of successive normal-to-normal interval differences; pSleep, night-time presumed sleep.

Supplementary Table S2. Burden of invalidated beats (ectopy and artifact) removed per recording segment during HRV pre-processing.

Segment	N	Total beats	PAC (SVEB)	PVC (VEB)	Artifact	Total invalidated
Visit 1	47	327 (197–671)	0.6 (0–6); 0.00% (0.00–0.02)	0.9 (0–17); 0.00% (0.00–0.05)	0.9 (0–12); 0.00% (0.00–0.05)	2.3 (0–20); 0.01% (0.00–0.06)
Visit 2	46	329 (255–443)	0.5 (0–5); 0.00% (0.00–0.02)	0.9 (0–19); 0.00% (0.00–0.06)	0.7 (0–9); 0.00% (0.00–0.02)	2.0 (0–19); 0.01% (0.00–0.06)
Home	47	314 (201–383)	0.6 (0–5); 0.00% (0.00–0.02)	0.2 (0–4); 0.00% (0.00–0.01)	0.3 (0–6); 0.00% (0.00–0.02)	1.1 (0–7); 0.00% (0.00–0.02)
pSleep 1	47	275 (210–373)	0.7 (0–8); 0.00% (0.00–0.03)	0.4 (0–10); 0.00% (0.00–0.04)	0.0 (0–1); 0.00% (0.00–0.00)	1.1 (0–10); 0.00% (0.00–0.04)
pSleep 2	47	276 (216–373)	1.0 (0–13); 0.00% (0.00–0.05)	0.4 (0–10); 0.00% (0.00–0.04)	0.2 (0–4); 0.00% (0.00–0.01)	1.6 (0–13); 0.01% (0.00–0.05)

For each beat type, cells show the number of invalidated beats and, after the semicolon, the percentage of total beats in the segment, both expressed as per-participant mean (range). 'Total beats' shows the mean (range) number of beats per segment. Ectopic and artefactual beats were manually marked invalid, and adjacent RR intervals were excluded from HRV calculation.

N, number of participants with valid data for the segment; PAC (SVEB), premature atrial complex / supraventricular ectopic beat; PVC (VEB), premature ventricular complex / ventricular ectopic beat; pSleep, night-time presumed sleep.
